# Online Learning for DNN Training: A Stochastic Block Adaptive Gradient Algorithm

**DOI:** 10.1155/2022/9337209

**Published:** 2022-06-02

**Authors:** Jianghui Liu, Baozhu Li, Yangfan Zhou, Xuhui Zhao, Junlong Zhu, Mingchuan Zhang

**Affiliations:** ^1^School of Information Engineering, Henan University of Science and Technology, Luoyang 471023, China; ^2^Internet of Things & Smart City Innovation Platform, Zhuhai Fudan Innovation Institute, Zhuhai, China

## Abstract

Adaptive algorithms are widely used because of their fast convergence rate for training deep neural networks (DNNs). However, the training cost becomes prohibitively expensive due to the computation of the full gradient when training complicated DNN. To reduce the computational cost, we present a stochastic block adaptive gradient online training algorithm in this study, called SBAG. In this algorithm, stochastic block coordinate descent and the adaptive learning rate are utilized at each iteration. We also prove that the regret bound of $O\left(\sqrt{T}\right)$ can be achieved via SBAG, in which *T* is a time horizon. In addition, we use SBAG to train ResNet-34 and DenseNet-121 on CIFAR-10, respectively. The results demonstrate that SBAG has better training speed and generalized ability than other existing training methods.

## 1. Introduction

Benefitting from a great many data samples and complex training model, deep learning has gained great interest in recent years and has been applied in resource allocation [1–4], signal estimation [5, 6], computer vision [7–9], and so on. However, the computing cost is very high in the training process of deep learning, which needs large amounts of training data and iteration update to obtain good model parameters. It is key to speed up model training process and improve model performance. Therefore, besides proposing new training architecture [10], designing an effective training algorithm is also important. This study focuses on the design of efficient training algorithms for deep neural networks (DNNs). In fact, many questions in practice can be modeled to be an optimization problem in general [11–13], which can be solved by employing gradient-based methods. The stochastic gradient descent (SGD) method is an effective optimization algorithm [14]. Moreover, it is easy to implement because of its simplicity and is frequently used in the training process of DNN.

Although the simplicity of stochastic gradient descents, the problem of slow convergence rate always exists. The same learning rate is not suitable for all parameter updates across the training process, especially in the case of sparse training data. For this reason, a number of training methods are presented to address this issue, for instance, AdaGrad [15], RMSProp[16], AdaDelta [17], and Adam [18]. These methods are referred as Adam-type algorithms since the adaptive learning rates are employed. Further, Adam has attained the most wide application in many deep learning training tasks, such as optimization of convolutional neural networks and recurrent neural networks [19, 20]. Despite its popularity, Adam incurs the convergence issue. For this reason, AMSGrad [21] was presented by introducing a nonincreasing learning rate. Besides, the learning rates of the Adam algorithm are either too big or too small, which results in poor generalization performance. To avoid the learning rate of extreme cases, a variant of Adam, Padam [22], was presented via employing a partial adaptive parameter *p*. SWATS [23] used the switch method from Adam to SGD. AdaBound [24] limited the learning rate to a dynamic bound over time at each iteration.

In deep learning, gradient-based methods are used to optimize the model parameter, which needs to calculate the gradients of all coordinates in decision vectors at each iteration, and a huge number of data and complex model lead to expensive computation cost. Randomized block coordinate descent is an efficient method for high-dimensional optimization problem and has been successfully utilized in the large-scale problem generated in machine learning [25]. It divides the set of variables into different blocks and carries out a gradient update step on a selected block coordinates randomly at each iteration, while holding the remaining ones fixed. In this way, the computational expense of each iteration can be effectively reduced.

In this study, we propose a stochastic block adaptive gradient online learning (SBAG) algorithm to rapidly train DNN, which incorporates an adaptive learning rate and stochastic block coordinate approach to improve the generalization ability and computation cost. Our key contributions are as follows:

• We present the SBAG algorithm based on the stochastic block coordinate descent method and AdaBound optimization algorithm to solve high-dimensional optimization problems.
(i) We provide the theoretical analysis on the convergence for SBAG. Moreover, we show that SBAG is convergent in the convex setting under common assumptions and its regret is bounded by $O\left(\sqrt{T}\right)$, where *T* is the time horizon.
(ii) We demonstrate the performance of SBAG on a public dataset. The simulation results show that the algorithm takes lesser time to achieve the best accuracy on the training set and test set, and it outperforms other methods.

The rest of this study is arranged as follows. In the next two sections, we will review the extant literature and introduce related background. In Section 4, we will present SBAG in detail. In Sections 5 and 6, we will describe our convergence analysis and performance evaluation. Finally, we present the conclusion of this paper in Section 7.

## 2. Related Work

SGD is one of the most popular algorithms used in DNN because of its implementation easily. However, it has the same learning rate for all parameters updated at each iteration across the training process, and the parameters are updated to the same extent no matter how different the feature frequencies are, which consequently results in slow convergence rate and poor performance. Hence, some variants of SGD were proposed to improve its convergence rate by either making the learning rate adaptive or using historical gradient information for descent direction. Ghadimi et al. [26] used the Heavy-ball method to combine one-order historical gradients and current gradients for updates. Sutskever et al. [27] presented Nesterov's accelerated gradient (NAG) method. Duchi et al. [15] proposed AdaGrad that first used an adaptive learning rate, whereas AdaGrad's performance is worse in the case of dense gradients because all historical gradients are used in the updates, and this limitation is more severe when dealing with high-dimensional data in deep learning. Hinton [16] proposed RMSProp, which utilizes an exponential moving average to solve the problem that the learning rate drops sharply in AdaGrad. Zeiler [17] proposed AdaDelta, which prevents learning rate decay and gradient disappearance over time. In fact, further research was to combine adaptive learning rate with historical gradient information, such as those used in Adam [18] and AMSGrad [21]. Moreover, Adam has a good convergence rate in many scenarios. However, it was found that Adam may not converge in the later stage of the training process on account of oscillated learning rate. Reddi et al. [21] presented AMSGrad, but the result of the experiments was not much better than Adam. In general, Adam-type algorithms have better performance on convergence, but often do not work well as SGD in out of sample. To address this issue, Keskar and Socher [23] proposed the SWATS algorithm. SWATS utilizes Adam to learn in the early part of the training and switches to SGD in the later stage of the training. In this case, it enjoys the quick convergence rate of Adam and the good performance of SGD, but the switching time is difficult to determine in practice. Huang et al. [28] presented NosAdam increasing the effect of past gradients on parameter update to avoid trapping in local or divergence. Nevertheless, it depends a lot on the initial conditions. Padam [22] introduced a parameter *p* making the level of adaptivity of the update process controlled. Luo et al. [24] proposed the AdaBound algorithm, which provides a dynamic bound for learning rate, and AdaBound is evaluated on a public dataset and is shown to converge as fast as Adam and perform as well as SGD. However, the aforementioned methods need to calculate all coordinates of gradients in decision vectors at each iteration, and computation cost will be aggravated due to the high-dimensional data and complex model structure.

The randomized block coordinate descent method is a powerful and effective approach for the high-dimensional optimization problem. It employs randomized strategies to pick a block of variables to update per iteration. For general gradient descent algorithms, all the coordinates of gradient vector should be calculated each time. One can easily observe that this will incur significant computing cost when dealing with high-dimensional data. In contrast, the randomized block coordinate method only calculates one block coordinate of gradient vector, which is considered as the descent direction. In particular, the randomized block method selects a coordinate based on probability *p* and updates the responding decision variable according to its descent direction. In addition, other coordinates of decision vector remain the same as the last time. Although the randomized block coordinate method can save significant computing cost for the learner, especially in optimization problems with high dimension data, it uses the fixed learning rate that scales the entries of gradient equally, and an adaptive learning rate has not been applied in this method.

Compared with the current work, we combine the randomized block coordinate descent method with an adaptive learning rate in this study. At each iteration, a part of gradient vectors is picked randomly, and the corresponding decision vectors are updated. In this way, the gradient is then calculated based on the chosen block coordinates instead of full gradients. Moreover, the extreme learning rates are restricted to a suitable range. Our method not only enjoys good generalization performance but also saves computation cost.

## 3. Preliminaries

In this section, we first introduce the optimization problem in detail. Then, we begin with the background about the randomized block coordinate method.

### 3.1. The Online Optimization Problem

In this work, the analysis of sequence iteration optimization problem is based on the online learning framework, which can be seen as a trade-off between a learner (the algorithm) and an opponent. In such an online convex setting, the learner selects a decision point **x**_*t*_ ∈ *𝒳* produced by the algorithm per time step *t*, *t*=1,…, *T*, and *𝒳* is a convex and compact subset of *ℝ*^*n*^. At the same time, the opponent responds to the decision of the learner with a loss function *f*_*t*_, which is convex and unknown in advance, and the algorithm suffers a loss *f*_*t*_(**x**_*t*_). Repeating the process, we have a sequence of loss functions {*f*_1_(**x**_1_), *f*_2_(**x**_2_),…, *f*_*t*_(**x**_*t*_)} where *f*_*t*_ : *𝒳*⟶*ℝ*, and they vary with time *t*. In general, the online learner's prediction problem can be represented as follows:

(1) $$\begin{array}{c} \underset{x\in X}{\min}\sum_{t=1}^{T}f_{t}\left(x\right). \end{array}$$

For online learning tasks, the goal is to optimize the regret *R*_*T*_ of the online learner's predictions against the optimal decision in hindsight, which is defined as the difference in the total sum of loss functions ∑_*t*=1_^*T*^*f*_*t*_(**x**_*t*_) after performing online learning over *T* rounds and its minimum value ∑_*t*=1_^*T*^*f*_*t*_(**x**^*∗*^) in the deterministic decision point **x**^*∗*^. In particular, we define the regret in the following:

(2) $$\begin{array}{c} R_{T}=\sum_{t=1}^{T}f_{t}\left(x_{t}\right)-\sum_{t=1}^{T}f_{t}\left(x^{*}\right), \end{array}$$

where **x**^*∗*^≔argmin_**x**∈*𝒳*_ *f*_*t*_(**x**), *t*=1,2,…, *T*. It is desired that if the regret of online optimization algorithm is a sublinear function of *T*, which suggests lim_*T*⟶*∞*_*R*_*T*_/*T*=0, then, on average, the online learner executes just and the fixed optimal decision afterwards. In other words, the proposed algorithm converges when its *R*_*T*_ is bounded. Throughout this study, the diameter of convex compact set *𝒳* is assumed to be bounded and ‖∇*f*_*t*_(**x**_*t*_)‖ is bounded for all *t*=1,2,…, *T*. Hereafter, ‖*·*‖ denotes the *ℓ*_2_ norm.

### 3.2. Relevant Definitions

Now, we will describe the relevant definitions that are used in the next sections.

**Definition 1 .**

A function *f*(*·*) : *𝒳*⟶*ℝ* is *L*-Lipschitz, where *L* is Lipschitz constant, and *L* > 0; if ∀**x**, **y** ∈ *𝒳*,

(3) $$\begin{array}{c} \left|f\left(x\right)-f\left(y\right)\right|\leq L\left\|x-y\right\|. \end{array}$$

**Definition 2 .**

(Equation (3.2) of Section 3 in [29]) A function *f*(*·*) : *𝒳*⟶*ℝ* is convex and differentiable where *𝒳* is a convex set; if ∀**x**, **y** ∈ *𝒳*,

(4) $$\begin{array}{c} f\left(y\right)\geq f\left(x\right)+\langle \nabla f\left(x\right),\left(y-x\right)\rangle . \end{array}$$

**Definition 3 .**

A function *f*(*·*) : *𝒳*⟶*ℝ* is *σ*-strongly convex and differentiable, *σ* > 0, and if ∀**x**, **y** ∈ *𝒳*,

(5) $$\begin{array}{c} f\left(y\right)\geq f\left(x\right)+\langle \nabla f\left(x\right),\left(y-x\right)\rangle +\frac{\sigma }{2}{\left\|x-y\right\|}^{2}. \end{array}$$

## 4. SBAG Algorithm and Assumptions

This section presents the proposed algorithm, followed by the common assumptions for convergence analysis of the algorithm.

### 4.1. Algorithm Design

In this study, we develop the high-dimensional online learning problems and aim to solve the optimization problem (1) by incorporating the stochastic block coordination method and adaptive learning rate. Because the dimensionality *n* of the decision variable **x** is high, the computing cost of the gradients is prohibitive. In addition, the tuning of the learning rate is challenging. For these reasons, a stochastic block coordinate adaptive optimization algorithm, dubbed SBAG, is proposed for settling the online problem (1). In our algorithm, the objective functions at different times satisfy some conditions, which are displayed in Assumption 1.

SBAG is described in Algorithm 1, whose input includes **x**_1_=0, **m**_1_=0, and **v**_1_=0. The parameters of SBAG are *β*_1*t*_ ∈ [0,1), *β*_1_≜*β*_11_, *β*_2_ ∈ [0,1), and $\alpha_{t}=1/\sqrt{t}$, where *t*=1,2,…, *T*. At each round *t*, a *n* order diagonal matrix *M*_*t*_ is generated and includes random variables {*w*_*t*,*i*_} with ℙ(*w*_*t*,*i*_=0)≔1 − *p*_*t*_ and ℙ(*w*_*t*,*i*_=1)≔*p*_*t*_, for *t*=0,1,…, *T* and *i*=1,…, *n*. In particular, the gradient **d**_*t*_ is computed as follows.

(6) $$\begin{array}{c} d_{t}=M_{t}\nabla f_{t}\left(x_{t}\right), \end{array}$$

where *M*_*t*_≜diag{**w**_*t*_}=diag{*w*_*t*,1_, *w*_*t*,2_,…, *w*_*t*,*n*_}, and elements of **w**_*t*_ consist of 0 and 1. When *w*_*t*,*i*_=1, it means that the *i*th coordinate of decision vector is selected to calculate the gradient at time *t*. From (6), one can observe that the computation cost is greatly reduced at each iteration. In addition, let ℋ^*t*^ denotes the *σ*− algebra, which means ℋ^*t*^ consists of all variables before time *t*. More explicitly, ℋ^*t*^={*M*_1_, *M*_2_,…, *M*_*t*−1_}.

By Using **d**_*t*_, one and second momentum terms **m**_*t*_ and **v**_*t*_ are obtained as follows, respectively.

(7) $$\begin{array}{c} m_{t}=\beta_{1t}m_{t-1}+\left(1-\beta_{1t}\right)d_{t}, \end{array}$$

(8) $$\begin{array}{c} v_{t}=\beta_{2}v_{t-1}+\left(1-\beta_{2}\right)d_{t}^{2}. \end{array}$$

Furthermore, SBAG introduces a bound of learning rate as follows:

(9) $$\begin{array}{c} {\tilde{\mu }}_{t}=\text{Clip}\left\{\frac{\alpha }{\sqrt{V_{t}}},\mu_{\text{low}}\left(t\right),\mu_{\text{upp}}\left(t\right)\right\}, \end{array}$$

where each element of the learning rate $\alpha /\sqrt{V_{t}}$ is clipped to constrain in an internal at time *t*, and the upper and lower bounds of the interval are *μ*_low_(*t*) and *μ*_upp_(*t*), respectively. That is, the output of equation (9) is constrained in [*μ*_low_(*t*), *μ*_upp_(*t*)], and the technique was also used in [23, 24]. Moreover, let

(10) $$\begin{array}{c} \mu_{t}=\frac{{\tilde{\mu }}_{t}}{\sqrt{t}}. \end{array}$$

Then, SBAG updates **x**_*t*+1_ as follows:

(11) $$\begin{array}{c} x_{t+1}=\Pi_{X,\text{diag}\left\{\mu_{t}^{-1}\right\}}\left(x_{t}-\mu_{t}{}^{\circ}m_{t}\right), \end{array}$$

where ° is the coordinate-wise product operator. Furthermore, the projection step of equation (11) is equivalent to the following:

(12) $$\begin{array}{c} x_{t+1}=\text{arg}\min_{x\in X}\left\|\mu_{t}^{-1/2}{}^{\circ}\left[x-\left(x_{t}-\mu_{t}{}^{\circ}m_{t}\right)\right]\right\|. \end{array}$$

### 4.2. Assumptions

Before presenting the convergence analysis of SBAG, we will now introduce the below common assumptions.

**Assumption 1 .**

Loss functions {*f*_1_(**x**), *f*_2_(**x**),…, *f*_*t*_(**x**)}, where *t*=1,2,…, *T*, are convex, differentiable, and *L*-Lipschitz over *𝒳*.

**Assumption 2 .**

In this study, *𝒳* is a bounded feasible set; *i.e.*, ‖**x**_*i*_ − **x**_*j*_‖ ≤ *B*_*∞*_, where *i*, *j* ∈ {1,2,…, *T*} and *B*_*∞*_ > 0.

**Assumption 3 .**

In this study, ‖∇*f*_*t*_(**x**_*t*_)‖ is bounded for all *t*=1,2,…, *T* over *𝒳*; *i.e.*, ‖∇*f*_*t*_(**x**_*t*_)‖ ≤ *C*_*∞*_, where *C*_*∞*_ > 0.

Assumptions 1–3 are some standard assumptions in the literature, for example [18, 21, 24]. In addition, the convergence of SBAG is analyzed based on these assumptions in the following.

## 5. Convergence Analysis

Now, we will analyze the convergence of SBAG. We consider the regret, equation (2), in the online optimization problem (a typical scenario). The proposed algorithm generates the gradient **d**_*t*_ with probability *p*_*t*_ at time *t*. Therefore, **d**_*t*_ is a random variable. Moreover, **x**_*t*_ is calculated by **d**_*t*_ and **x**_*t*−1_ at time *t*. According to the knowledge of probability and statistics, the expectation should be considered when the variable is randomized. Therefore, we define the regret of SBAG as follows:

(13) $$\begin{array}{c} {\hat{R}}_{T}=\sum_{t=1}^{T}\left[E\left[f_{t}\left(x_{t}\right)\right]-f_{t}\left(x^{*}\right)\right]. \end{array}$$

From the convexity of *f*_*t*_, it follows that

(14) $$\begin{array}{c} f_{t}\left(x_{t}\right)-f_{t}\left(x^{*}\right)\leq \nabla f_{t}{\left(x_{t}\right)}^{⊤}\left(x_{t}-x^{*}\right). \end{array}$$

Moreover, by the definition of matrix *M*_*t*_, we know that *M*_*t*_ is a sparse matrix. Therefore, applying equation (14) leads to

(15) $$\begin{array}{c} f_{t}\left(x_{t}\right)-f_{t}\left(x^{*}\right)\leq \left\|M_{t}\nabla f_{t}{\left(x_{t}\right)}^{⊤}\left(x_{t}-x^{*}\right)\right\|\\ +\nabla f_{t}{\left(x_{t}\right)}^{⊤}\left(x_{t}-x^{*}\right)=\left\|d_{t}^{⊤}\left(x_{t}-x^{*}\right)\right\|+\nabla f_{t}{\left(x_{t}\right)}^{⊤}\left(x_{t}-x^{*}\right). \end{array}$$

Taking conditional expectation (conditioned on ℋ^*t*^) on both sides of equation (15), it implies that

(16) $$\begin{array}{c} E\left[f_{t}\left(x_{t}\right)|{ℋ}^{t}\right]-E\left[f_{t}\left(x^{*}\right)|{ℋ}^{t}\right]\leq E\left[\left\|d_{t}^{⊤}\left(x_{t}-x^{*}\right)\right\||{ℋ}^{t}\right]+E\left[\nabla f_{t}{\left(x_{t}\right)}^{⊤}\left(x_{t}-x^{*}\right)|{ℋ}^{t}\right]. \end{array}$$

By equation (1.1f) of Section 4 in [30], and taking unconditional expectation for equation (16), it follows that

(17) $$\begin{array}{c} E\left(\left[f_{t}\left(x_{t}\right)\right]-f_{t}\left(x^{*}\right)\right]\leq E\left[\left\|d_{t}^{⊤}\left(x_{t}-x^{*}\right)\right\|\right]+E\left[\nabla f_{t}{\left(x_{t}\right)}^{⊤}\left(x_{t}-x^{*}\right)\right]. \end{array}$$

From equations (13) and (17), the following equation holds

(18) $$\begin{array}{c} {\hat{R}}_{T}=\sum_{t=1}^{T}E\left[\left\|d_{t}^{⊤}\left(x_{t}-x^{*}\right)\right\|\right]+\sum_{t=1}^{T}E\left[\nabla f_{t}{\left(x_{t}\right)}^{⊤}\left(x_{t}-x^{*}\right)\right]. \end{array}$$

To get the bound of ${\hat{R}}_{T}$, we should consider the two terms on the right side of equation (18). Thus, we first propose the following lemmata to estimate term ∑_*t*=1_^*T*^*𝔼*[‖**d**_*t*_^⊤^(**x**_*t*_ − **x**^*∗*^)‖].

**Lemma 1 .**

If Assumptions 1to 3 are satisfied, sequences {**x**_*t*_}, {**m**_*t*_}, and {**v**_*t*_} are generated by SBAG with *t* ∈ {1,2,…, *T*}. Moreover, *𝒳* is a convex and compact set. *β*_1_≔*β*_11_, *β*_1*t*_ ≤ *β*_1(*t* − 1)_ ≤ *β*_1_, and $\beta_{1}/\sqrt{\beta_{2}}\leq 1$ for *t*=1,…, *T*. In addition, suppose *μ*_low_(*t*+1) ≥ *μ*_low_(*t*) ≥ 0, *μ*_upp_(*t*+1) ≤ *μ*_upp_(*t*), and lim_*t*⟶*∞*_*μ*_low_(*t*)=lim_*t*⟶*∞*_*μ*_upp_(*t*)=*α*, where *α* > 0. Let *L*_*∞*_ ≔*μ*_low_(1), *U*_*∞*_ ≔*μ*_upp_(1), and *p*_*t*_ ∈ [*p*_min_, *p*_max_]. Then, we have the following relation:

(19) $$\begin{array}{c} \sum_{t=1}^{T}E\left[{\left\|\mu_{t}^{1/2}{}^{\circ}m_{t}\right\|}^{2}\right]\leq \frac{p_{\text{max}}\beta_{1}U_{\infty }\left(2\sqrt{T}-1\right)}{1-\beta_{1}}\sum_{i=1}^{n}{\left\|d_{1:T,i}\right\|}^{2}. \end{array}$$

**Proof**

From equations (9) and (10), it follows that

(20) $$\begin{array}{c} \sqrt{t}{\left\|\mu_{t}\right\|}_{\infty }\leq \mu_{\text{upp}}\left(t\right)\leq \mu_{\text{upp}}\left(1\right)≔U_{\infty }, \end{array}$$

and

(21) $$\begin{array}{c} \sqrt{t}{\left\|\mu_{t}\right\|}_{\infty }\geq \mu_{\text{low}}\left(t\right)\geq \mu_{\text{low}}\left(1\right)≔L_{\infty }. \end{array}$$

From equations (20) and (21), and by property of expectation, it can be verified that

(22) $$\begin{array}{c} \sum_{t=1}^{T}E\left[{\left\|\mu_{t}^{1/2}{}^{\circ}m_{t}\right\|}^{2}\right]\leq \sum_{t=1}^{T}E\left[{\left\|m_{t}\right\|}^{2}\frac{U_{\infty }}{\sqrt{t}}\right]=U_{\infty }\sum_{t=1}^{T}\frac{p_{t}{\left\|m_{t}\right\|}^{2}}{\sqrt{t}}\leq p_{\text{max}}U_{\infty }\sum_{t=1}^{T}\frac{{\left\|m_{t}\right\|}^{2}}{\sqrt{t}},\\ =p_{\text{max}}U_{\infty }\sum_{t=1}^{T-1}\frac{{\left\|m_{t}\right\|}^{2}}{\sqrt{t}}+p_{\text{max}}U_{\infty }\sum_{i=1}^{n}\frac{m_{T,i}^{2}}{\sqrt{T}}. \end{array}$$

Plugging equation (7) into equation (22), it yields

(23) $$\begin{array}{c} \sum_{t=1}^{T}E\left[{\left\|\mu_{t}^{1/2}{}^{\circ}m_{t}\right\|}^{2}\right]\leq p_{\text{max}}U_{\infty }\sum_{t=1}^{T-1}\frac{{\left\|m_{t}\right\|}^{2}}{\sqrt{t}}+\frac{p_{\text{max}}U_{\infty }}{\sqrt{T}}\underset{\left(a\right)}{\underset{︸}{\sum_{i=1}^{n}{\left(\sum_{j=1}^{T}\left(1-\beta_{1j}\right)\prod_{k=1}^{T-j}\beta_{1\left(T-k+1\right)}d_{j,i}\right)}^{2}}}. \end{array}$$

By Cauchy–Schwarz inequality, we further bound the term (a) of equation (23) and have

(24) $$\begin{array}{c} \left(a\right)\leq \sum_{i=1}^{n}\left[\sum_{j=1}^{T}\prod_{k=1}^{T-j}\beta_{1\left(T-k+1\right)}\right]\left[\sum_{j=1}^{T}\prod_{k=1}^{T-j}\beta_{1\left(T-k+1\right)}d_{j,i}^{2}\right]\leq \sum_{i=1}^{n}\left(\sum_{j=1}^{T}\beta_{1}^{T-j}\right)\left(\sum_{j=1}^{T}\beta_{1}^{T-j}d_{j,i}^{2}\right)\\ \leq \frac{1}{1-\beta_{1}}\sum_{i=1}^{n}\sum_{j=1}^{T}\beta_{1}^{T-j}d_{j,i}^{2}. \end{array}$$

The second inequality of equation (24) follows from the fact *β*_1*k*_ ≤ *β*_1_ for all *k* ∈ {1,…, *T*}. In addition, the third inequality of equation (24) is due to the inequality ∑_*j*=1_^*T*^*β*_1_^*T*−*j*^ ≤ 1/1 − *β*_1_. Moreover, plugging equation (24) into equation (23) leads to

(25) $$\begin{array}{c} \sum_{t=1}^{T}E\left[{\left\|\mu_{t}^{1/2}{}^{\circ}m_{t}\right\|}^{2}\right]\leq p_{\text{max}}U_{\infty }\sum_{t=1}^{T-1}\frac{{\left\|m_{t}\right\|}^{2}}{\sqrt{t}}\\ +\frac{p_{\text{max}}U_{\infty }}{\left(1-\beta_{1}\right)\sqrt{T}}\sum_{i=1}^{n}\sum_{j=1}^{T}\beta_{1}^{T-j}d_{j,i}^{2}\\ \leq \frac{p_{\text{max}}U_{\infty }}{1-\beta_{1}}\sum_{t-1}^{T}\frac{1}{\sqrt{t}}\sum_{i=1}^{n}\sum_{j=1}^{t}\beta_{1}^{t-j}d_{j,i}^{2}\\ \leq \frac{p_{\text{max}}U_{\infty }}{1-\beta_{1}}\sum_{i=1}^{n}\sum_{t-1}^{T}\frac{1}{\sqrt{t}}\sum_{j=1}^{t}\beta_{1}^{t-j}d_{j,i}^{2}\\ \leq \frac{p_{\text{max}}U_{\infty }}{1-\beta_{1}}\sum_{i=1}^{n}\sum_{t-1}^{T}d_{t,i}^{2}\sum_{j=1}^{t}\frac{\beta_{1}^{t-j}}{\sqrt{j}}\\ \leq \frac{p_{\text{max}}\beta_{1}U_{\infty }}{1-\beta_{1}}\sum_{i=1}^{n}\sum_{t-1}^{T}d_{t,i}^{2}\sum_{j=1}^{t}\frac{1}{\sqrt{j}}. \end{array}$$

Moreover, since $\sum_{t=1}^{T}1/\sqrt{t}\leq 1+\int_{1}^{T}1/\sqrt{t}dt=2\sqrt{T}-1$, and by equation (25), it follows that

(26) $$\begin{array}{c} \sum_{t=1}^{T}E\left[{\left\|\mu_{t}^{1/2}{}^{\circ}m_{t}\right\|}^{2}\right]\leq \frac{p_{\text{max}}\beta_{1}U_{\infty }\left(2\sqrt{T}-1\right)}{1-\beta_{1}}\sum_{i=1}^{n}\sum_{t-1}^{T}d_{t,i}^{2}\\ \leq \frac{p_{\text{max}}\beta_{1}U_{\infty }\left(2\sqrt{T}-1\right)}{1-\beta_{1}}\sum_{i=1}^{n}{\left\|d_{1:T,i}\right\|}^{2}. \end{array}$$

Therefore, the proof of Lemma 1 is completed. Next, we introduce Lemma 2 to estimate the term ∑_*t*=1_^*T*^*𝔼*[‖**d**_*t*_^⊤^(**x**_*t*_ − **x**^*∗*^)‖].

**Lemma 2 .**

If Assumptions 1to 3 are satisfied, sequences {**x**_*t*_}, {**m**_*t*_}, and {**v**_*t*_} are generated by SBAG with *t* ∈ {1,2,…, *T*}. Moreover, *𝒳* is a convex and compact set. *β*_1_ ≔*β*_11_, *β*_1*t*_ ≤ *β*_1(*t* − 1)_ ≤ *β*_1_, and $\beta_{1}/\sqrt{\beta_{2}}\leq 1$, for *t*=1,…, *T*. In addition, suppose *μ*_low_(*t*+1) ≥ *μ*_low_(*t*) ≥ 0, *μ*_upp_(*t*+1) ≤ *μ*_upp_(*t*), and lim_*t*⟶*∞*_*μ*_low_(*t*)=lim_*t*⟶*∞*_*μ*_upp_(*t*)=*α*, where *α* > 0. Let *L*_*∞*_≔*μ*_low_(1), *U*_*∞*_≔*μ*_upp_(1), and *p*_*t*_ ∈ [*p*_min_, *p*_max_]. Then, we have the following:

(27) $$\begin{array}{c} \sum_{t=1}^{T}E\left[\left\|d_{t}^{⊤}\left(x_{t}-x^{*}\right)\right\|\right]\leq \frac{B_{\infty }^{2}L_{\infty }\sqrt{T}}{2\left(1-\beta_{1}\right)p_{\min}}+\frac{\beta_{1}B_{\infty }^{2}L_{\infty }}{2\left(1-\beta_{1}\right)\left(1-\lambda \right)p_{\min}}\\ +\frac{p_{\text{max}}\beta_{1}U_{\infty }\left(2\sqrt{T}-1\right)}{{\left(1-\beta_{1}\right)}^{2}}\sum_{i=1}^{n}{\left\|d_{1:T,i}\right\|}^{2}. \end{array}$$

**Proof**

Let **x**^*∗*^≔argmin_**x**∈*𝒳*_ *f*_*t*_(**x**) with *t*=1,2,…, *T*. By equations (11) and (12), the following equation holds

(28) $$\begin{array}{c} x_{t+1}=\Pi_{X,\text{diag}\left\{\mu_{t}^{-1}\right\}}\left(x_{t}-\mu_{t}{}^{\circ}m_{t}\right)\\ =\text{arg}\min_{x\in X}\left\|\mu_{t}^{-1/2}{}^{\circ}\left[x-\left(x_{t}-\mu_{t}{}^{\circ}m_{t}\right)\right]\right\|. \end{array}$$

Using Lemma 3 of [31], it can be proved that

(29) $$\begin{array}{c} {\left\|\mu_{t}^{-1/2}{}^{\circ}\left(x_{t+1}-x^{*}\right)\right\|}^{2}\leq {\left\|\mu_{t}^{-1/2}{}^{\circ}\left(\left(x_{t}-\mu_{t}{}^{\circ}m_{t}\right)-x^{*}\right)\right\|}^{2}\\ ={\left\|\mu_{t}^{-1/2}{}^{\circ}\left(x_{t}-x^{*}\right)\right\|}^{2}-2m_{t}^{⊤}\left(x_{t}-x^{*}\right)\\ +{\left\|\mu_{t}^{1/2}{}^{\circ}m_{t}\right\|}^{2}. \end{array}$$

Substituting equation (7) into equation (29) yields

(30) $$\begin{array}{c} {\left\|\mu_{t}^{-1/2}{}^{\circ}\left(x_{t+1}-x^{*}\right)\right\|}^{2}\leq {\left\|\mu_{t}^{-1/2}{}^{\circ}\left(x_{t}-x^{*}\right)\right\|}^{2}+{\left\|\mu_{t}^{1/2}{}^{\circ}m_{t}\right\|}^{2}\\ -2{\left[\beta_{1t}m_{t-1}+\left(1-\beta_{1t}\right)d_{t}\right]}^{⊤}\left(x_{t}-x^{*}\right),\\ ={\left\|\mu_{t}^{-1/2}{}^{\circ}\left(x_{t}-x^{*}\right)\right\|}^{2}+{\left\|\mu_{t}^{1/2}{}^{\circ}m_{t}\right\|}^{2}\\ -2\beta_{1t}m_{t-1}^{⊤}\left(x_{t}-x^{*}\right)-2\left(1-\beta_{1t}\right)d_{t}^{⊤}\left(x_{t}-x^{*}\right). \end{array}$$

Rearranging the terms of equation (30), and by *β*_1*t*_ ≤ *β*_1(*t* − 1)_, it follows that

(31) $$\begin{array}{c} d_{t}^{⊤}\left(x_{t}-x^{*}\right)\leq \frac{{\left\|\mu_{t}^{-1/2}{}^{\circ}\left(x_{t}-x^{*}\right)\right\|}^{2}}{2\left(1-\beta_{1t}\right)}+\frac{{\left\|\mu_{t}^{1/2}{}^{\circ}m_{t}\right\|}^{2}}{2\left(1-\beta_{1t}\right)}\\ -\frac{\beta_{1t}m_{t-1}^{⊤}\left(x_{t}-x^{*}\right)}{1-\beta_{1t}}-\frac{{\left\|\mu_{t}^{-1/2}{}^{\circ}\left(x_{t+1}-x^{*}\right)\right\|}^{2}}{2\left(1-\beta_{1t}\right)}\\ \leq \frac{{\left\|\mu_{t}^{-1/2}{}^{\circ}\left(x_{t}-x^{*}\right)\right\|}^{2}-{\left\|\mu_{t}^{-1/2}{}^{\circ}\left(x_{t+1}-x^{*}\right)\right\|}^{2}}{2\left(1-\beta_{1t}\right)}+\frac{{\left\|\mu_{t}^{1/2}{}^{\circ}m_{t}\right\|}^{2}}{2\left(1-\beta_{1t}\right)}+\frac{\beta_{1t}m_{t-1}^{⊤}\left(x_{t}-x^{*}\right)}{1-\beta_{1t}}. \end{array}$$

Applying Young's inequality and the Cauchy–Schwarz inequality into equation (31) leads to

(32) $$\begin{array}{c} d_{t}^{⊤}\left(x_{t}-x^{*}\right)\leq \frac{{\left\|\mu_{t}^{-1/2}{}^{\circ}\left(x_{t}-x^{*}\right)\right\|}^{2}-{\left\|\mu_{t}^{-1/2}{}^{\circ}\left(x_{t+1}-x^{*}\right)\right\|}^{2}}{2\left(1-\beta_{1t}\right)}\\ +\frac{{\left\|\mu_{t}^{1/2}{}^{\circ}m_{t}\right\|}^{2}}{2\left(1-\beta_{1t}\right)}+\frac{\beta_{1t}}{2\left(1-\beta_{1t}\right)}{\left\|\mu_{t}^{1/2}{}^{\circ}m_{t-1}\right\|}^{2}+\frac{\beta_{1t}}{2\left(1-\beta_{1t}\right)}{\left\|\mu_{t}^{-1/2}{}^{\circ}\left(x_{t}-x^{*}\right)\right\|}^{2}. \end{array}$$

Summing equation (32) over *t* ∈ {1,2,…, *T*} and taking expectation on the obtained relation imply that

(33) $$\begin{array}{c} \sum_{t=1}^{T}E\left[\left\|d_{t}^{⊤}\left(x_{t}-x^{*}\right)\right\|\right]\leq E\left[\frac{{\left\|\mu_{t}^{-1/2}{}^{\circ}\left(x_{t}-x^{*}\right)\right\|}^{2}-{\left\|\mu_{t}^{-1/2}{}^{\circ}\left(x_{t+1}-x^{*}\right)\right\|}^{2}}{2\left(1-\beta_{1t}\right)}\right]\\ +\frac{1}{2\left(1-\beta_{1t}\right)}E\left[{\left\|\mu_{t}^{1/2}{}^{\circ}m_{t}\right\|}^{2}+{\left\|\mu_{t}^{1/2}{}^{\circ}m_{t-1}\right\|}^{2}\right]+\frac{1}{2\left(1-\beta_{1t}\right)}E\left[\beta_{1t}{\left\|\mu_{t}^{-1/2}{}^{\circ}\left(x_{t}-x^{*}\right)\right\|}^{2}\right]\\ \leq \text{E}\left[\frac{{\left\|\mu_{t}^{-1/2}{}^{\circ}\left(x_{t}-x^{*}\right)\right\|}^{2}-{\left\|\mu_{t}^{-1/2}{}^{\circ}\left(x_{t+1}-x^{*}\right)\right\|}^{2}}{2\left(1-\beta_{1}\right)}\right]+\frac{1}{2\left(1-\beta_{1}\right)}E\left[{\left\|\mu_{t}^{1/2}{}^{\circ}m_{t}\right\|}^{2}+{\left\|\mu_{t}^{1/2}{}^{\circ}m_{t-1}\right\|}^{2}\right]\\ +\frac{1}{2\left(1-\beta_{1}\right)}E\left[\beta_{1t}{\left\|\mu_{t}^{-1/2}{}^{\circ}\left(x_{t}-x^{*}\right)\right\|}^{2}\right]. \end{array}$$

By Lemma 1 and equation (33), it follows from that

(34) $$\begin{array}{c} \sum_{t=1}^{T}E\left[\left\|d_{t}^{⊤}\left(x_{t}-x^{*}\right)\right\|\right]\leq \sum_{t=1}^{T}E\left[\frac{{\left\|\mu_{t}^{-1/2}{}^{\circ}\left(x_{t}-x^{*}\right)\right\|}^{2}}{2\left(1-\beta_{1}\right)}\right]\leq -\sum_{t=1}^{T}E\left[\frac{{\left\|\mu_{t}^{-1/2}{}^{\circ}\left(x_{t+1}-x^{*}\right)\right\|}^{2}}{2\left(1-\beta_{1}\right)}\right]\\ +\sum_{t=1}^{T}\frac{E\left[\beta_{1t}{\left\|\mu_{t}^{-1/2}{}^{\circ}\left(x_{t}-x^{*}\right)\right\|}^{2}\right]}{2\left(1-\beta_{1}\right)}+\frac{p_{\text{max}}\beta_{1}U_{\infty }\left(2\sqrt{T}-1\right)}{{\left(1-\beta_{1}\right)}^{2}}\sum_{i=1}^{n}{\left\|d_{1:T,i}\right\|}^{2}\\ \leq E\left[\sum_{i=1}^{n}\frac{\mu_{1,i}^{-1}{\left(x_{1,i}-x_{i}^{*}\right)}^{2}}{2\left(1-\beta_{1}\right)}\right]+E\left[\sum_{t=2}^{T}\sum_{i=1}^{n}\frac{\mu_{t,i}^{-1}{\left(x_{t,i}-x_{i}^{*}\right)}^{2}-\mu_{t-1,i}^{-1}{\left(x_{t,i}-x_{i}^{*}\right)}^{2}}{2\left(1-\beta_{1}\right)}\right]\\ +\sum_{t=1}^{T}\sum_{i=1}^{n}\frac{E\left[\beta_{1t}\mu_{t,i}^{-1}{\left(x_{t,i}-x_{i}^{*}\right)}^{2}\right]}{2\left(1-\beta_{1}\right)}+\frac{p_{\text{max}}\beta_{1}U_{\infty }\left(2\sqrt{T}-1\right)}{{\left(1-\beta_{1}\right)}^{2}}\sum_{i=1}^{n}{\left\|d_{1:T,i}\right\|}^{2}. \end{array}$$

Since $\mu_{t}={\tilde{\mu }}_{t}/\sqrt{t}=\alpha /\sqrt{t{\hat{v}}_{t}}$ and ${\hat{v}}_{t}=\max\left\{v_{t},v_{t-1}\right\}$, we have 0 < *μ*_*t*_ ≤ *μ*_*t*−1_. Therefore, we further obtain *μ*_*t*_^−1^ ≥ *μ*_*t*−1_^−1^. Then, from equation (34), it can be proved that

(35) $$\begin{array}{c} \sum_{t=1}^{T}E\left[\left\|d_{t}^{⊤}\left(x_{t}-x^{*}\right)\right\|\right]\leq E\left[\sum_{i=1}^{n}\frac{\mu_{1,i}^{-1}{\left(x_{1,i}-x_{i}^{*}\right)}^{2}}{2\left(1-\beta_{1}\right)}\right]\\ +E\left[\sum_{t=2}^{T}\sum_{i=1}^{n}\frac{\mu_{t,i}^{-1}{\left(x_{t,i}-x_{i}^{*}\right)}^{2}-\mu_{t-1,i}^{-1}{\left(x_{t,i}-x_{i}^{*}\right)}^{2}}{2\left(1-\beta_{1}\right)}\right]+\sum_{t=1}^{T}\sum_{i=1}^{n}\frac{E\left[\beta_{1t}\mu_{t,i}^{-1}{\left(x_{t,i}-x_{i}^{*}\right)}^{2}\right]}{2\left(1-\beta_{1}\right)}\\ +\frac{\left(2\sqrt{T}-1\right)p_{\max^{2}}U_{\infty }C_{\infty }^{2}}{1-\beta_{1}}\leq E\left[\sum_{i=1}^{n}\frac{\mu_{T,i}^{-1}{\left(x_{T,i}-x_{i}^{*}\right)}^{2}}{2\left(1-\beta_{1}\right)}\right]+\sum_{t=1}^{T}\sum_{i=1}^{n}\frac{E\left[\beta_{1t}\mu_{t,i}^{-1}{\left(x_{t,i}-x_{i}^{*}\right)}^{2}\right]}{2\left(1-\beta_{1}\right)}\\ +\frac{p_{\text{max}}\beta_{1}U_{\infty }\left(2\sqrt{T}-1\right)}{{\left(1-\beta_{1}\right)}^{2}}\sum_{i=1}^{n}{\left\|d_{1:T,i}\right\|}^{2}. \end{array}$$

Applying Assumption 2 and property of expectation yields

(36) $$\begin{array}{c} \sum_{t=1}^{T}E\left[\left\|d_{t}^{⊤}\left(x_{t}-x^{*}\right)\right\|\right]\leq \frac{B_{\infty }^{2}}{2\left(1-\beta_{1}\right)p_{\min}}\sum_{i=1}^{n}\mu_{T,i}^{-1}\\ +\frac{B_{\infty }^{2}}{2\left(1-\beta_{1}\right)p_{\min}}\sum_{t=1}^{T}\sum_{i=1}^{n}\beta_{1t}\mu_{t,i}^{-1}+\frac{p_{\text{max}}\beta_{1}U_{\infty }\left(2\sqrt{T}-1\right)}{{\left(1-\beta_{1}\right)}^{2}}\sum_{i=1}^{n}{\left\|d_{1:T,i}\right\|}^{2}\\ \leq \frac{B_{\infty }^{2}L_{\infty }\sqrt{T}}{2\left(1-\beta_{1}\right)p_{\min}}+\frac{\beta_{1}B_{\infty }^{2}L_{\infty }}{2\left(1-\beta_{1}\right)\left(1-\lambda \right)p_{\min}}+\frac{p_{\text{max}}\beta_{1}U_{\infty }\left(2\sqrt{T}-1\right)}{{\left(1-\beta_{1}\right)}^{2}}\sum_{i=1}^{n}{\left\|d_{1:T,i}\right\|}^{2}. \end{array}$$

Therefore, the proof of Lemma 2 is completed. Next, we estimate the last term in (18).

**Lemma 3 .**

If Assumptions 1to 3 are satisfied, sequences {**x**_*t*_}, {**m**_*t*_}, and {**v**_*t*_} are generated by SBAG with *t* ∈ {1,2,…, *T*}. Moreover, *𝒳* is a convex and compact set. *β*_1_≔*β*_11_, *β*_1*t*_ ≤ *β*_1_ and $\beta_{1}/\sqrt{\beta_{2}}\leq 1$, for *t*=1,…, *T*. In addition, suppose *μ*_low_(*t*+1) ≥ *μ*_low_(*t*) ≥ 0, *μ*_upp_(*t*+1) ≤ *μ*_upp_(*t*), and lim_*t*⟶*∞*_*μ*_low_(*t*)=lim_*t*⟶*∞*_*μ*_upp_(*t*)=*α*, where *α* > 0. Let *L*_*∞*_≔ *μ*_low_(1) and *U*_*∞*_≔*μ*_upp_(1). Then, we attain the following inequality:

(37) $$\begin{array}{c} \sum_{t=1}^{T}E\left[\nabla f_{t}{\left(x_{t}\right)}^{⊤}\left(x_{t}-x^{*}\right)\right]\leq \frac{B_{\infty }^{2}L_{\infty }\sqrt{T}}{2\left(1-\beta_{1}\right)}\\ +\frac{\beta_{1}B_{\infty }^{2}L_{\infty }}{2\left(1-\beta_{1}\right)\left(1-\lambda \right)}+\frac{\beta_{1}U_{\infty }\left(2\sqrt{T}-1\right)}{{\left(1-\beta_{1}\right)}^{2}}\sum_{i=1}^{n}{\left\|d_{1:T,i}\right\|}^{2}. \end{array}$$

**Proof**

For the original full gradient, we have *𝔼*[∇*f*_*t*_(**x**_*t*_)]=∇*f*_*t*_(**x**_*t*_). Let **m**_*t*_′≔*β*_1*t*_**m**_*t*−1_′+(1 − *β*_1*t*_)∇*f*_*t*_(**x**_*t*_), **v**_*t*_′=*β*_2_**v**_*t*−1_′+(1 − *β*_2_)∇*f*_*t*_^2^(**x**_*t*_), and $\mu_{t}^{\prime }={\tilde{\mu }}_{t}^{\prime }/\sqrt{t}$, which are generated by AdaBound [24].

The proof of Lemma 3 is similar to that of Theorem 4 in [24]. Starting with the following inequality implies

(38) $$\begin{array}{c} \sum_{t=1}^{T}E\left[\left\|\nabla f_{t}{\left(x_{t}\right)}^{⊤}\left(x_{t}-x^{*}\right)\right\|\right]\leq \frac{{\left\|{\mu^{\prime }}_{t}^{-1/2}{}^{\circ}\left(x_{t}-x^{*}\right)\right\|}^{2}-{\left\|{\mu^{\prime }}_{t}^{-1/2}{}^{\circ}\left(x_{t+1}-x^{*}\right)\right\|}^{2}}{2\left(1-\beta_{1}\right)}\\ +\frac{{\left\|{\mu^{\prime }}_{t}^{1/2}{}^{\circ}m_{t}^{\prime }\right\|}^{2}+{\left\|{\mu^{\prime }}_{t}^{1/2}{}^{\circ}m_{t-1}^{\prime }\right\|}^{2}}{2\left(1-\beta_{1}\right)}+\frac{\beta_{1t}{\left\|{\mu^{\prime }}_{t}^{-1/2}{}^{\circ}\left(x_{t}-x^{*}\right)\right\|}^{2}}{2\left(1-\beta_{1}\right)}\\ \leq \frac{{\left\|{\mu^{\prime }}_{t}^{-1/2}{}^{\circ}\left(x_{t}-x^{*}\right)\right\|}^{2}-{\left\|{\mu^{\prime }}_{t}^{-1/2}{}^{\circ}\left(x_{t+1}-x^{*}\right)\right\|}^{2}}{2\left(1-\beta_{1}\right)}+\frac{\beta_{1t}{\left\|{\mu^{\prime }}_{t}^{-1/2}{}^{\circ}\left(x_{t}-x^{*}\right)\right\|}^{2}}{2\left(1-\beta_{1}\right)}\\ +\frac{\beta_{1}U_{\infty }\left(2\sqrt{T}-1\right)}{{\left(1-\beta_{1}\right)}^{2}}\sum_{i=1}^{n}{\left\|d_{1:T,i}\right\|}^{2}\leq \frac{B_{\infty }^{2}L_{\infty }\sqrt{T}}{2\left(1-\beta_{1}\right)}+\frac{\beta_{1}B_{\infty }^{2}L_{\infty }}{2\left(1-\beta_{1}\right)\left(1-\lambda \right)}+\frac{\beta_{1}U_{\infty }\left(2\sqrt{T}-1\right)}{{\left(1-\beta_{1}\right)}^{2}}\sum_{i=1}^{n}{\left\|d_{1:T,i}\right\|}^{2}. \end{array}$$

Therefore, the proof of Lemma 3 is finished.

To attain the bound of regret ${\hat{R}}_{T}$ in equation (18), we establish Theorem 1 as follows.

**Theorem 1 .**

Suppose that Assumptions 1to 3 are satisfied, and sequences {**x**_*t*_}, {**m**_*t*_}, and {**v**_*t*_} are generated by SBAG with *t* ∈ {1,2,…, *T*}. Moreover, *𝒳* is a convex and compact set. *β*_1_≔*β*_11_, *β*_1*t*_ ≤ *β*_1_, and $\beta_{1}/\sqrt{\beta_{2}}\leq 1$ for *t*=1,…, *T*. In addition, suppose *μ*_low_(*t*+1) ≥ *μ*_low_(*t*) ≥ 0, *μ*_upp_(*t*+1) ≤ *μ*_upp_(*t*), and lim_*t*⟶*∞*_*μ*_low_(*t*)=lim_*t*⟶*∞*_*μ*_upp_(*t*)=*α*, where *α* > 0. Let *L*_*∞*_≔*μ*_low_(1), *U*_*∞*_≔*μ*_upp_(1), and *p*_*t*_ ∈ [*p*_min_, *p*_max_]. We obtain the bound of regret as follows:

(39) $$\begin{array}{c} {\hat{R}}_{T}\leq \frac{\left(1+p_{\min}\right)B_{\infty }^{2}L_{\infty }\sqrt{T}}{2\left(1-\beta_{1}\right)p_{\min}}+\frac{\beta_{1}\left(1+p_{\min}\right)B_{\infty }^{2}L_{\infty }}{2\left(1-\beta_{1}\right)\left(1-\lambda \right)p_{\min}}\\ +\frac{\left(1+p_{\max}\right)\beta_{1}U_{\infty }\left(2\sqrt{T}-1\right)}{{\left(1-\beta_{1}\right)}^{2}}\sum_{i=1}^{n}{\left\|d_{1:T,i}\right\|}^{2}. \end{array}$$

**Proof**

Applying lemmata 1, 2, and 3 into (18) yields

(40) $$\begin{array}{c} {\hat{R}}_{T}\leq \frac{B_{\infty }^{2}L_{\infty }\sqrt{T}}{2\left(1-\beta_{1}\right)p_{\min}}+\frac{\beta_{1}B_{\infty }^{2}L_{\infty }}{2\left(1-\beta_{1}\right)\left(1-\lambda \right)p_{\min}}\\ +\frac{p_{\max}\beta_{1}U_{\infty }\left(2\sqrt{T}-1\right)}{{\left(1-\beta_{1}\right)}^{2}}\sum_{i=1}^{n}{\left\|d_{1:T,i}\right\|}^{2}+\frac{B_{\infty }^{2}L_{\infty }\sqrt{T}}{2\left(1-\beta_{1}\right)}\\ +\frac{\beta_{1}B_{\infty }^{2}L_{\infty }}{2\left(1-\beta_{1}\right)\left(1-\lambda \right)}+\frac{\beta_{1}U_{\infty }\left(2\sqrt{T}-1\right)}{{\left(1-\beta_{1}\right)}^{2}}\sum_{i=1}^{n}{\left\|d_{1:T,i}\right\|}^{2}\\ =\frac{\left(1+p_{\min}\right)B_{\infty }^{2}L_{\infty }\sqrt{T}}{2\left(1-\beta_{1}\right)p_{\min}}+\frac{\beta_{1}\left(1+p_{\min}\right)B_{\infty }^{2}L_{\infty }}{2\left(1-\beta_{1}\right)\left(1-\lambda \right)p_{\min}}\\ +\frac{\left(1+p_{\max}\right)\beta_{1}U_{\infty }\left(2\sqrt{T}-1\right)}{{\left(1-\beta_{1}\right)}^{2}}\sum_{i=1}^{n}{\left\|d_{1:T,i}\right\|}^{2}. \end{array}$$

Therefore, we complete the proof of Theorem 1.

From Theorem 1, we obtain ${\text{lim}}_{T⟶\infty }{\hat{R}}_{T}/T=0$. This suggests that SBAG is convergent. In addition, the bound of regret ${\hat{R}}_{T}$ is $O\left(\sqrt{T}\right)$; i.e., given some accuracy *ε*, it requires an order of *𝒪*(1/*ε*^2^) iterations at least to achieve the given accuracy.

## 6. Performance Evaluation

In this section, we perform our experiments on a public dataset to evaluate the performance of algorithm objectively. We consider the machine learning problem, multi-classification tasks taking advantage of the DNN for the experiments.

### 6.1. Setup

To assess our SBAG algorithm, we research the performance on the classification task problem. We use the CIFAR-10 [32] dataset for our experiments, which is widely used for classification problem. It consists of 10 classes and 50000 training samples and 10000 test samples.

For the experiments, we use the convolutional neural network to solve classification tasks on the CIFAR-10 image dataset, which has a good effect on image classification and object recognition, and specifically implement ResNet-34 [33] and DenseNet-121 [34].

### 6.2. Parameters

To study the performance of our proposed algorithm, we compare SBAG with SGD [14], AdaGrad [15], and AdaBound [24]. The hyper-parameters of these algorithms are initialized as follows.

For SGD, the scale of the learning rate is selected from the set {100,10,1,0.1, 0.01}. AdaGrad uses the initialized learning rate set {5e − 2,1e − 2,5e − 3,1e − 3,5e − 4}, and the value 0 is set for the initial accumulator value of AdaGrad. The value of hyper-parameters of AdaBound is set the same as Adam. We directly use the initialized hyper-parameter values of AdaBound in our algorithm. In addition, we set the probability of choosing a coordinate from these values in the set {0.10%, 0.50%, 1.00%, 5.00%, 10.00%, 50.00%}.

In addition, we define the dynamic bound functions following with [24] for our simulation experiments, i.e.,

(41) $$\begin{array}{c} \mu_{\text{low}}\left(t\right)=0.1-\frac{0.1}{\left(1-\beta_{2}\right)t+1}, \end{array}$$

and

(42) $$\begin{array}{c} \mu_{\text{upp}}\left(t\right)=0.1+\frac{0.1}{\left(1-\beta_{2}\right)t}. \end{array}$$

### 6.3. Results

**Figure 1:**
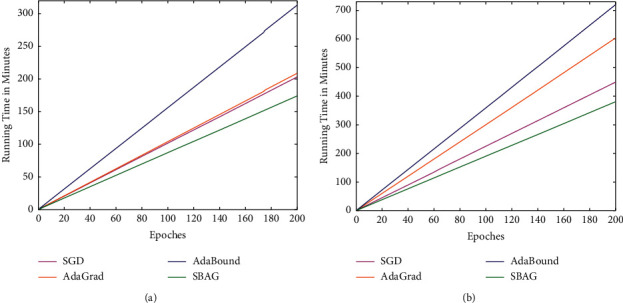
Running time with epochs for ResNet-34 and DenseNet-121 on CIFAR-10: a comparative summary. (a) Runtime for ResNet-34. (b) Runtime for DenseNet-121.

We take account of the image multi-class classification problem on the CIFAR-10 dataset using ResNet-34 and DenseNet-121 and run 200 epoch in this experiment. First, we operate a group of experiments with epochs and runtime for ResNet-34 and DenseNet-121 on CIFAR-10. The findings of experiments are reported in Figure 1, and when completing the same number of iterations of 200 epochs, our method takes the least time, and the AdaBound spends the most time. The main reason is that only several blocks of coordinates are calculated in the gradient descent process for our algorithm at each iteration *t*, while the compared algorithms calculate the full gradients at each iteration. Moreover, AdaBound combines the first- and second-order momentum, while SGD and AdaGrad only use first-order gradients; thus, SGD and AdaGrad incur less time than AdaBound. The same results can be seen for the DenseNet-121 in Figure 1(b).

**Figure 2:**
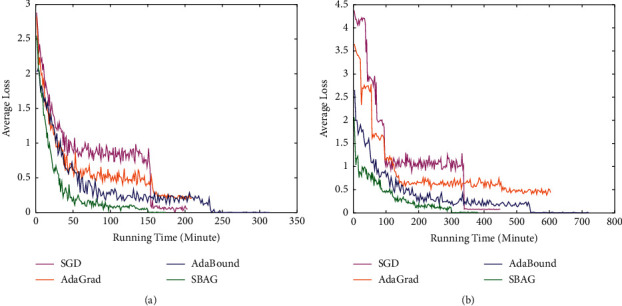
Loss with running time for ResNet-34 and DenseNet-121 on CIFAR-10: a comparative summary. (a) Loss for ResNet-34. (b) Loss for DenseNet-121.

We present another group of experiments with average loss and running time, which are executed for ResNet-34 and DenseNet-121 on CIFAR-10. The findings are shown in Figure 2. At about 150 epochs, SGD has the biggest average loss than others and decreases sharply after that time, while the average loss of SBAG is smaller compared with others and reaches the minimum value in the shortest running time finally. The reason for fast descent rate of SBAG is due to the randomized block method, which chooses one block coordinate of decision vector to calculate the gradient. In other words, SBAG calculates more samples than other compared algorithms in the same running time. Therefore, the convergence of SBAG is verified by the findings presented in Figure 2.

**Figure 3:**
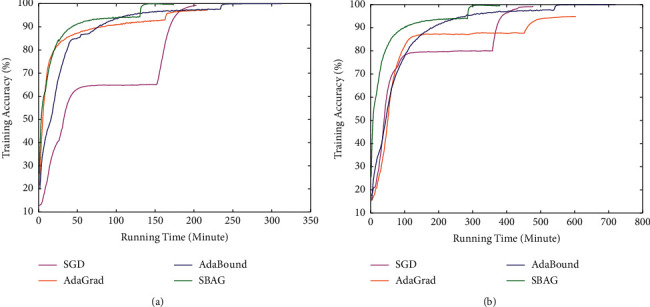
Training accuracy with running time for ResNet-34 and DenseNet-121 on CIFAR-10: a comparative summary. (a) Training accuracy for ResNet-34. (b) Training accuracy for DenseNet-121.

**Figure 4:**
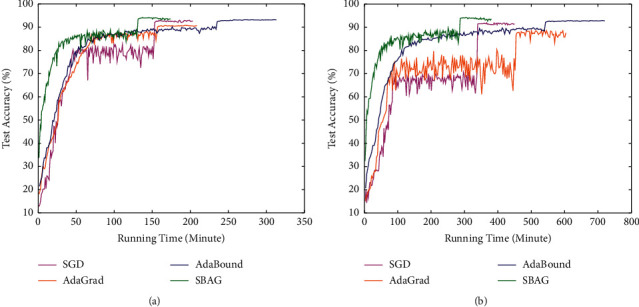
Test accuracy with running time for ResNet-34 and DenseNet-121 on CIFAR-10: a comparative summary. (a) Test accuracy for ResNet-34. (b) Test accuracy for DenseNet-121.

In Figures 3 and 4, the training and test accuracy with running time of four algorithms are evaluated. As we can see, in about 150 epochs, AdaBound achieves the highest accuracy, and AdaGrad and our algorithm almost have the same accuracy of 92.36% and 93.99%. As the running time goes, the AdaBound and SBAG have the accuracy of 99.96% and 99.93%, respectively. The similar results can be seen on the DenseNet-121. In a word, SBAG works well on training or test set, and at the same time, it has the good generalization ability on both ResNet-34 and DenseNet-121.

From the experiments above, we observe that the SBAG shows a very good performance on both ResNet-34 and DenseNet-121. It incurs less computation cost for each iteration in experiments, which is consistent with theory.

## 7. Conclusion

In this study, we proposed a randomized block adaptive gradient online learning algorithm. The proposed algorithm, SBAG, is designed to reduce the gradient computation cost of high-dimensional decision vector. The convergence analysis of SBAG and evaluations on CIFAR-10 demonstrated that the regret bound of SBAG is $O\left(\sqrt{T}\right)$ when loss functions are convex and achieved significant computation cost savings, without adversely affecting the performance of the optimizer. In the same 200 epochs, the proposed algorithm has the least running time and tightly less in average loss in the end. The accuracy of training sample for ResNet-34 and DenseNet-121 is 99.93% and 99.72%, slightly less compared with that of 99.96% of AdaBound, but our method reaches the highest accuracy on the test sample than AdaBound, SGD, and AdaGrad; i.e., SBAG is the fastest in four methods, and the curves are milder than SGD.

## Figures and Tables

**Algorithm 1:**
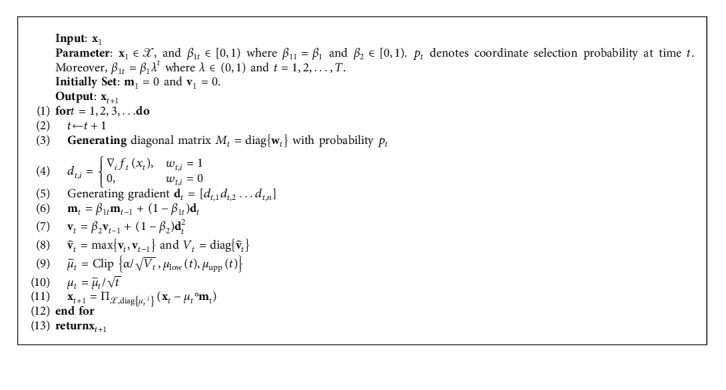
SBAG.

## Data Availability

The data that support the findings of this study are CIFAR-10, which is available from [32].
